# Cost-Effectiveness of Combined Sexual and Injection Risk Reduction Interventions among Female Sex Workers Who Inject Drugs in Two Very Distinct Mexican Border Cities.

**DOI:** 10.1371/journal.pone.0147719

**Published:** 2016-02-18

**Authors:** Jose L. Burgos, Thomas L. Patterson, Joshua S. Graff-Zivin, James G. Kahn, M. Gudelia Rangel, M. Remedios Lozada, Hugo Staines, Steffanie A. Strathdee

**Affiliations:** 1 University of California San Diego, Department of Medicine, Division of Global Public Health, La Jolla, California, United States of America; 2 Universidad Autonoma de Baja California, Facultad de Medicina y Psicología, Tijuana, Baja California, México; 3 University of California San Diego, Department of Psychiatry, La Jolla, California, United States of America; 4 University of California San Diego, School of Global Policy and Strategy, La Jolla, California, United States of America; 5 University of California San Francisco, Department of Epidemiology and Biostatistics, Philip R. Lee Institute for Health Policy Studies, Global Health Sciences, San Francisco, California, United States of America; 6 Secretaria de Salud de México, Comision de Salud Fronteriza Mexico-Estados Unidos Sección México, Tijuana, Baja California, México; 7 Instituto de Servicios de Salud Pública del Estado de Baja California, Mexicali, Baja California, Mexico; 8 Universidad Autonoma de Ciudad Juarez, Facultad de Medicina, Ciudad Juárez, Chihuahua, México; David Geffen School of Medicine at UCLA, UNITED STATES

## Abstract

**Background:**

We evaluated the cost-effectiveness of combined single session brief behavioral intervention, either didactic or interactive (Mujer Mas Segura, MMS) to promote safer-sex and safer-injection practices among female sex workers who inject drugs (FSW-IDUs) in Tijuana (TJ) and Ciudad-Juarez (CJ) Mexico. Data for this analysis was obtained from a factorial RCT in 2008–2010 coinciding with expansion of needle exchange programs (NEP) in TJ, but not in CJ.

**Methods:**

A Markov model was developed to estimate the incremental cost per quality adjusted life year gained (QALY) over a lifetime time frame among a hypothetical cohort of 1,000 FSW-IDUs comparing a less intensive didactic vs. a more intensive interactive format of the MMS, separately for safer sex and safer injection combined behavioral interventions. The costs for antiretroviral therapy was not included in the model. We applied a societal perspective, a discount rate of 3% per year and currency adjusted to US$2014. A multivariate sensitivity analysis was performed. The combined and individual components of the MMS interactive behavioral intervention were compared with the didactic formats by calculating the incremental cost-effectiveness ratios (ICER), defined as incremental unit of cost per additional health benefit (e.g., HIV/STI cases averted, QALYs) compared to the next least costly strategy. Following guidelines from the World Health Organization, a combined strategy was considered highly cost-effective if the incremental cost per QALY gained fell below the gross domestic product per capita (GDP) in Mexico (equivalent to US$10,300).

**Findings:**

For CJ, the mixed intervention approach of interactive safer sex/didactic safer injection had an incremental cost-effectiveness ratio (ICER) of US$4,360 ($310–$7,200) per QALY gained compared with a dually didactic strategy. Using the dually interactive strategy had an ICER of US$5,874 ($310–$7,200) compared with the mixed approach. For TJ, the combination of interactive safer sex/didactic safer injection had an ICER of US$5,921 ($104–$9,500) per QALY compared with dually didactic. Strategies using the interactive safe injection intervention were dominated due to lack of efficacy advantage. The multivariate sensitivity analysis showed a 95% certainty that in both CJ and TJ the ICER for the mixed approach (interactive safer sex didactic safer injection intervention) was less than the GDP per capita for Mexico. The dual interactive approach met this threshold consistently in CJ, but not in TJ.

**Interpretation:**

In the absence of an expanded NEP in CJ, the combined-interactive formats of the MMS behavioral intervention is highly cost-effective. In contrast, in TJ where NEP expansion suggests that improved access to sterile syringes significantly reduced injection-related risks, the interactive safer-sex combined didactic safer-injection was highly cost-effective compared with the combined didactic versions of the safer-sex and safer-injection formats of the MMS, with no added benefit from the interactive safer-injection component.

## Introduction

Female sex workers (FSWs) have increased vulnerability for HIV and other sexually transmitted infections (STIs) particularly in low and middle-income countries. Globally 15% of HIV infections are associated to occupational risks for FSWs and over 160,000 annual deaths due to HIV infection are estimated to affect FSWs.[1] The financial burden of the HIV epidemic in Mexico has increased significantly from US$278 million in 2007 to over US$1 billion annually in 2012.[2–4]

Evidence based HIV prevention behavioral interventions have been shown to be highly cost effective and to potentially save public health resources in low and middle-income countries such as Mexico.[3, 5, 6] FSWs that inject drugs (FSW-IDUs) represent a particular vulnerable group with dual HIV and sexually transmitted infection (STI) risks associated with unsafe sex and unsafe injection practices.[7]

The northern border Mexican cities of Tijuana and Ciudad Juarez are known as major international drug trafficking routes from Latin America to the United States, where a large proportion of the heroin entering the U.S passes through.[8, 9] Injection drug use among FSWs is estimated at 18% with an HIV incidence of 2 per 100 person-years among all FSWs in Mexican northern border cities.[10, 11] FSW-IDUs have an increased HIV prevalence of 12% compared to 6% among FSW who do not inject drugs and the likelihood of having at least any active STI among this vulnerable group is of 50% compared to 25% among non IDU-FSWs.[9, 12]

In contrast to an overall low HIV prevalence in Mexico estimated at 0.3%, the northern Mexican border region has experienced an escalating HIV epidemic and concurrent growth of the IDU population. Between Tijuana and Cd. Juarez, the IDU populations is estimated to be close to 20,000, and previous studies have estimated that approximately 1 in 116 persons between the ages of 15 and 49 in Tijuana were living with HIV[13]. In 2009 Mexico was awarded funds from the Global Fund for HIV, TB and Malaria to scale up HIV prevention programs, including the expansion of needle exchange programs (NEP) in northern Mexican border cities.[9]

In 2010 we reported findings on the cost-effectiveness analysis (CEA) based on a randomized controlled trial for the *Mujer Segura* (Safe woman [MS]) 30 minute brief behavioral intervention to increase condom negotiation skills and condom use with clients among FSWs in Tijuana and Cd. Juarez.[3, 10] This study showed a 40% effectiveness in reducing the combined HIV/STI incidence and to save public health financial resources by producing net cost-savings from averted costs associated with HIV infection.[3] However, among FSW-IDUs the MS intervention did not show any significant impact in reducing unsafe injection practices (e.g. receptive needle sharing) and in this subgroup it was not shown to be cost-effective.[3, 14]

**Fig 1:**
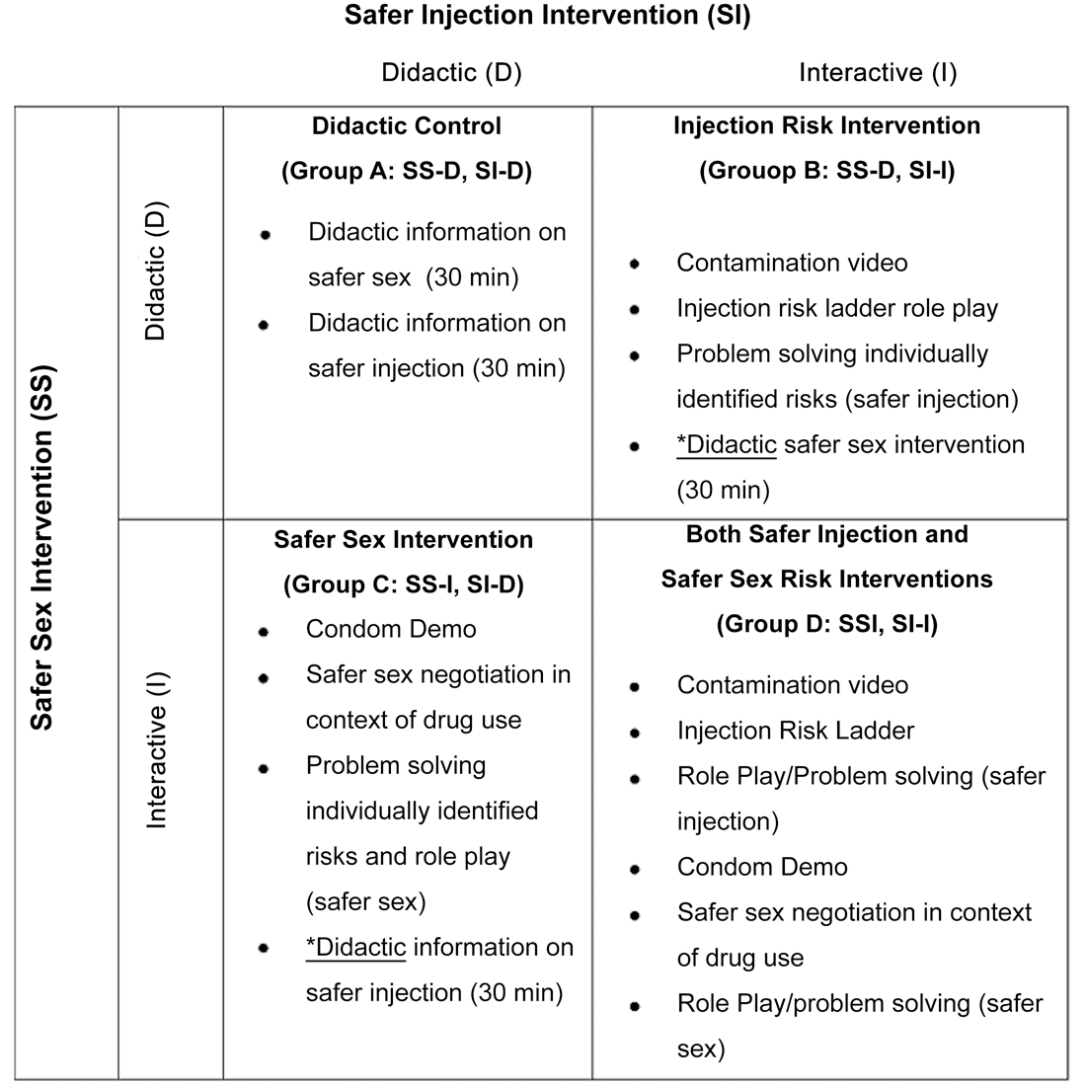
Mujer Mas Segura trial Design of 2x2 Factorial Trial to Simultaneously Evaluate Injection and Sexual Risk Reduction Interventions^1^. ^1^Adapted from Strathdee PloS ONE 2013.

Subsequent to findings from the MS randomized controlled trial (RCT), Drs. Strathdee, Patterson, and collaborators, developed a 60 minute theory based interactive behavioral intervention designed for Mexican FSW-IDUs called *Mujer Mas Segura* (MMS). This intervention incorporated principles of Motivational Interviewing, Social Cognitive Theory (SCT), and Theory of Reasoned Action to simultaneously reduce risky sexual practices with clients and risky injection.[15] Between October of 2008 and July 2010 584 adult HIV-negative FSW-IDU from Tijuana and Cd. Juarez were recruited to participate in a two-by-two factorial RCT to evaluate the impact of the interactive versus didactic approach, for both sexual and injection risks. [15] Participants received two interventions (safer sex [SS] and safer injection [SI]), each one either interactive (I; intervention) or didactic (D; control). Thus, one group received two interactive interventions (SS-I and SI-I), one group received two didactic interventions (SS-D and SI-D), and two groups received one interactive and one didactic. The 2x2 design permits comparison among these four groups. (See Fig 1)

At twelve months after receiving the MMS intervention, the interactive approach was superior for the safer sex intervention: FSW-IDUs assigned to SS-I had a 50% reduction in combined HIV/STI incidence compared to those assigned to SS-D. In Cd. Juarez, risky injection practices decreased significantly in SI-I compared with SI-D. In contrast, in Tijuana, the SI-I and SI-D groups reported similar reduction in syringe sharing. [16]

In this study, we assessed the cost-effectiveness of adding the interactive element for the safer sex and safer injection components of MMS among FSW-IDUs in Tijuana and Cd. Juarez.

## Methods

### Cost-effectiveness model

We developed a discrete Markov state transition model using TreeAge Pro Suite software (Williamstown, MA; TreeAge Software, Inc.) to estimate the cost-effectiveness of the combined components (didactic or interactive versions) of the MMS behavioral intervention. Our model represented the transition of 1,000 FSW-IDU through various health states, including uninfected and progressively more serious disease, based on data obtained from the MMS RCT. We conducted Monte Carlo simulations to calculate health outcomes (incident HIV infections or STIs, quality adjusted life years [QALYs], quality adjusted life expectancy [QALE]), and lifetime costs comparing a didactic vs. the interactive versions of each individual component of the MMS (e.g. interactive safer sex and safer injection components) and the combined interactive MMS behavioral intervention (Fig 1).[15, 17] The combined and individual components of the MMS interactive behavioral intervention were compared with the didactic formats by calculating the incremental cost-effectiveness ratios (ICER), defined as incremental unit of cost per additional health benefit (e.g. HIV/STI cases averted, QALYs) compared to the next least costly strategy.[18] We conducted the analysis separately for Tijuana and Cd. Juarez. We adopted a societal perspective, as recommended by the U.S. Panel of Cost-Effectiveness Analysis and the National Institute for Health and Clinical Excellence (NICE).[19, 20] FSWs characteristics from Tijuana and Cd. Juarez used in our model were based on participants from the MMS efficacy trial.[16] Costs are presented in 2014 US dollars and inflated according to the gross domestic product deflator[21] and currency exchange rates published by the Bank of Mexico.[22] Both cost and health outcomes were discounted at an annual rate of 3%. To determine cost-effectiveness, we followed guidelines from the World Health Organization and considered a strategy to be highly cost-effective if the cost per QALY gained fell below the gross domestic product per capita (GDP) in Mexico (US$10,300) and cost-effective if the cost per QALY gained was between 1 and 3 times the GDP per capita in Mexico, strategies with a cost per QALY gained greater than 3 times the GDP per capita were considered beyond an acceptable threshold to consider the intervention cost-effective.[22]

### Model specification

Our model considered the following strategies for comparison (see Fig 1): 1) Didactic versions of the MMS safer sex and safer injection components (i.e. SS-D, SI-D; assigned as the control group); 2) Interactive version of the safer injection and didactic version of the safer sex component (combined SS-D, SI-I); 3) Interactive version of the safer sex and didactic version of the safer injection components (SS-I, SI-D); 4) Interactive versions of both the MMS safer sex and safer injection components (SS-I, SI-I). Risks of HIV/ and Sexually transmitted infections (STIs) among FSW-IDUs were based on findings from the MMS efficacy trial[16] and were modeled as a sequence of annual transitions between seven mutually exclusive health states among FSW-IDUs. Initially 1,000 HIV negative FSW-IDU enter our model in one of three mutually exclusive health states: 1) No HIV or STI; 2) Non-ulcerative STI (i.e. *Neisseria gonorrhoeae*, *Chlamydia trachomatis*); 3) Ulcerative STI (i.e. syphilis infection). Additionally in each subsequent annual cycle, FSW-IDU can remain in, or transition between the initial three health states or to any of four additional health states: 4) HIV infection with no STI; 5) HIV and a non-ulcerative STI; 6) HIV and syphilis infection; 7) death.

To model HIV progression, we created a variable to track years since acquiring HIV infection adjusting for QALYs and increased risk of mortality according to projected reduction in CD4+ cells (Table 1).[23, 24]

**Table 1:** CD4+ counts and changes in mortality and health-related quality of life values used for the base case analysis.

CD4+ cells^a^	IRR^b^	QALY^c^
>450	1	0.98
400–450	1.41	0.96
300–399	1.45	0.94
200–299	1.66	0.94
100–199	2.59	0.87
50–99	4.63	0.81
0–49	11.63	0.79

^a^ Number of CD4+ cells per micro liter

^b^ Incidence rate ratio

^c^ Quality-adjusted life years

The expected effect from the MMS combined intervention was a reduction in HIV and STI’s from increased in condom use with clients (from the safe sex component of MMS) and reduction of unsafe injection practices (from the safe injection component of the MMS). The HIV risk due to unsafe injection practices was calculated using epidemiologic equations[25] based on HIV prevalence estimates among IDUs [16], reported unsafe injection practices among a cohort of 584 FSW-IDUs from Tijuana and Cd. Juarez[16] (data collected in the MMS efficacy trial). The comparison group for this analysis was the less intensive MMS that combined the didactic versions of the safer sex and safer injection modules.

### Variables and parameters

The values of the variables and parameters used in the model were derived from the Mujer Mas Segura RCT,[16] regional epidemiological, and when region specific data was unavailable, values from the medical and scientific literature were used as estimates (See Table 2). As described in previous reports,[1] the median age for FSWs recruited for the MMS randomized controlled trial was 33 years (interquartile range [IQR]: 27, 40), years of residency in the recruitment city was 22 years (IQR: 10, 32), 47% earned less than US$350 per month and a median number of unprotected sex acts with clients of 30 (IQR: 9, 60). To estimate HIV risk from unsafe injection practices, we used an HIV risk index that combines receptive needle sharing, sharing injection paraphernalia, regional prevalence rates among people who inject drugs (PWIDs), and transmission probabilities for HIV were calculated based on estimates for unsafe injection practices among FSW-IDUs (e.g. frequency of syringe or paraphernalia sharing) obtained from data collected in the MMS trial. [17, 25] The cost-effectiveness analysis considered HIV/STI risk for FSW-IDUs and did not account for HIV/STI cases averted among clients of FSW-IDUs.

**Table 2:** Model base case clinical values and ranges used for sensitivity analyses.

	Variable	Base Case (range)	Source
**Demographic**	Age of sex work initiation	19 (16–25)	^[^^15^^]^
**Epidemiological values**	HIV/STI combined incidence:		
	Among FSW-IDU in Tijuana^a^	64.3 (34.6–93.9)	^[^^16^^]^
	Among FSW-IDUs in Cd. Juarez^a^	66.1 (30.2–102.0)	^[^^16^^]^
	Calculated HIV injection risk:		
	HIV prevalence among used syringes^b^	12.3% (6–19%)	^[^^43^^,^ ^44^^]^
	Receptive sharing of contaminated syringe^b^	0.667	^[^^44^^,^ ^45^^]^
	Shared contaminated cotton/cooker/water^b^	0.0067	^[^^45^^]^
	Contaminated syringe cleaned with bleach^b^	0 (0–21)	^[^^45^^]^
**Utilities**	QALYs^c^ lost per STI episode	.005 (.003-.0089)	^[^^3^^]^

^a^Per 100 person years

^b^Per 100 exposures

^c^Quality Adjusted Life Years

Costs were obtained from accounting records from the Mujer Mas Segura RCT and regional economic indicators (e.g. personnel costs, counseling sessions, HIV/STI screening) using an ingredients approach (i.e. accounting for personnel time to deliver the intervention or supplies used for each intervention). For fixed costs we used a step down approach (i.e. based on average fair market costs).[26] Costs associated with treatment for STI were obtained from public government records and from the National HIV Program in Mexico (Centro Nacional para la Prevención y el Control del VIH y SIDA [CENSIDA]) website. [4, 27] All participants receiving the MMS intervention were screened for HIV and STIs (see Table 3). The costs for antiretroviral therapy was not included in the model considering the poor access to HIV care among these vulnerable population.[28]

**Table 3:** Costs associated with the Mujer Mas Segura intervention.

Costs		Value		Source	
Administrative personnel		$9,000^a^		[46, 47]	
Outreach workers		$9,600^b^		[46, 48]	
Counseling session (30 min)		$6.00		[46, 48]	
RN sample collection (15 min)		$3.00		[46, 48]	
Testing for Gc^c^/CT^d^		$22.00		MMS^e^, [49]	
RPR		$5.00		MMS^e^,[49]	
Rapid HIV test		$5.00		MMS^e^, [49]	
HIV confirmatory test		$56.00		MMS^e^, [49]	
Syringes, gloves, speculum, other		$2.00		MMS^e^, [49]	
Azitrhromycin 1 gr		$20.00		MMS^e^, [27]	
Ceftriaxone 250 mg		$12.70		MMS^e^, [27]	
Benzathine penicillin^f^		$10.00		MMS^e^, [27]	
Participant’s time		$6.29		[47]	
Space/Utilities/mileage		$7,900		MMS^e^	

^a^Administrative cost calculated at $7.25 per hour.

^b^Two outreach workers at $2.35 each per hour.

^c^Gonorrhea

^d^Chlamydia trachomatis

^e^Accounting records from the Mujer Mas Segura trial

^f^2.4 million units

### Sensitivity analysis

We conducted a probabilistic sensitivity analysis to assess uncertainly on all input values. For epidemiological inputs (HIV/STI incidence) we fitted beta distributions and gamma distributions for cost variables according to punctual and 95% confidence intervals (CI) [29–33] derived from the Mujer Mas Segura RCT and for the likely range of other inputs according to an extensive literature review. Distributions used in this analysis based on data from the MMS trial were created using Crystal Ball simulation Software (Oracle Corporation, Redwood Shores, CA). We performed a second-order Monte Carlo simulations to obtain 95% uncertainty ranges and to generate cost-effectiveness acceptability curves.

Clinical trial registration number: NCT00840658

## Results

### Base Case Results

For a hypothetical cohort of 1,000 FSWs the incremental cost for the interactive components of the Mujer Mas Segura intervention were $39 USD per person-year compared to the strategy combining the didactic versions of the combined MMS intervention in both cities.

#### Ciudad Juarez

Compared to the MMS combined didactic approach (SS-D, SI-D) participants in the group with interactive safe sex (SS-I) and didactic safer injection (SI-D) had increased Quality Adjusted Life Expectancy (QALE) of 64 days per person. The combined interactive format (SS-I, SI-I) added 47 days per person compared to the combined SS-I, SI-D. Compared with the didactic safer sex and didactic safer injection MMS, the incremental cost-effectiveness for the combined interactive safer sex/didactic safer injection intervention was US$4,360 per QALY compared to the combined SS-D, SI-D and US$5,874 per QALY for the MMS combined SS-I, SI-I components of the MMS intervention compared with the combined SS-I, SI-D. The strategy combining the didactic safer sex and interactive safer injection MMS (SS-D, SI-I) was dominated by the combined interactive safer sex and didactic safer injection strategy (SS-I, SI-D) and was excluded from the analysis.

#### Tijuana

In contrast, in Tijuana, all groups, including the control group (i.e. combined didactic safer sex and didactic safer injection combined MMS; SS-D, SI-D) showed a significant reduction on unsafe injection practices but no difference between the groups receiving the interactive format or didactic versions of the safer injection components of the MMS intervention, therefore, for Tijuana, the combined interactive components of the MMS intervention (SS-I, SI-I) did not show added benefits and therefore was dominated by the group receiving the combined interactive safer sex with the didactic safer injection components of the intervention (SS-I, SI-D). The interactive safer sex intervention averted 370 STI/HIV infections among a cohort of 1,000 FSWs over a lifetime-horizon and increased the quality-adjusted life expectancy (QALE) by 85 days. The incremental cost-effectiveness for the combined interactive safer sex and the didactic safer injection components of the intervention was US$5,921 per QALY gained.

### Multivariate Sensitivity Analysis

**Fig 2:**
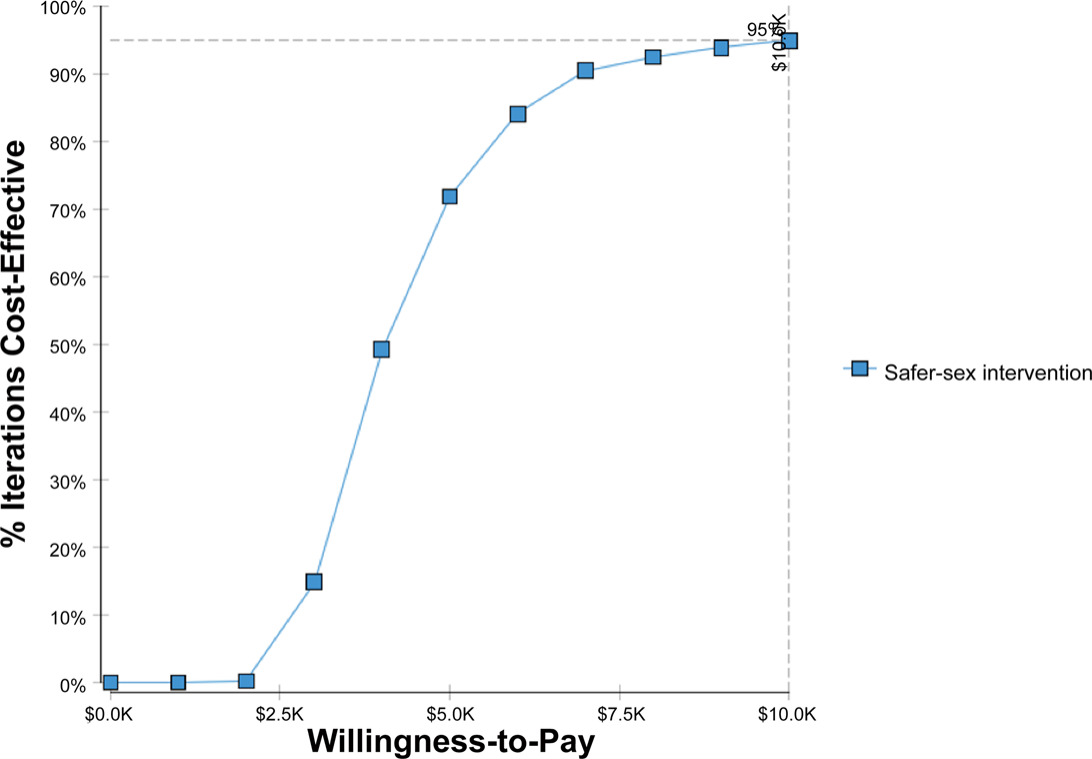
Cost-effectiveness acceptability curve for the combined interactive safer sex/ didactic safer injection intervention in Cd. Juarez.

**Fig 3:**
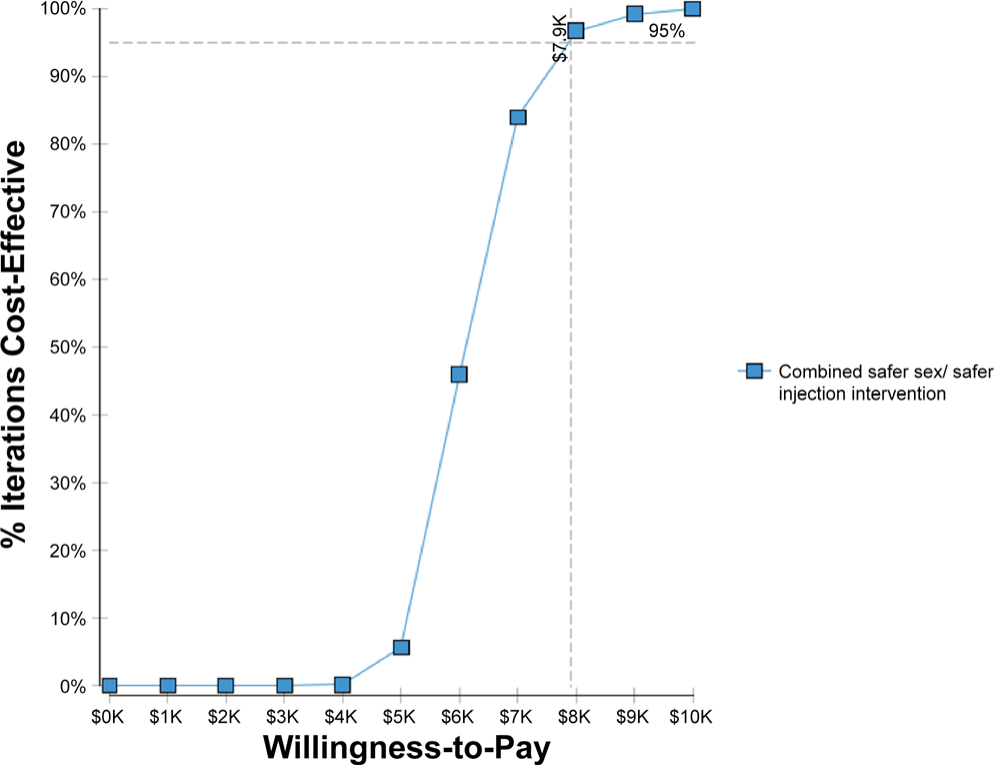
Cost-effectiveness acceptability curve for the combined interactive safer sex/Safer injection intervention for Ciudad Juarez.

For Ciudad Juarez the multivariate analysis showed 95% likelihood that the ICER for the combined interactive safer sex and didactic safer injection components would be less than 59% compared with the control group (didactic safer sex and safer injection combined MMS strategy and less than 47% of the GDP per capita in Mexico for the combined interactive components of the MMS intervention compared with the combined interactive safer sex/ didactic safer injection MMS (see Figs 2 and 3).

**Fig 4:**
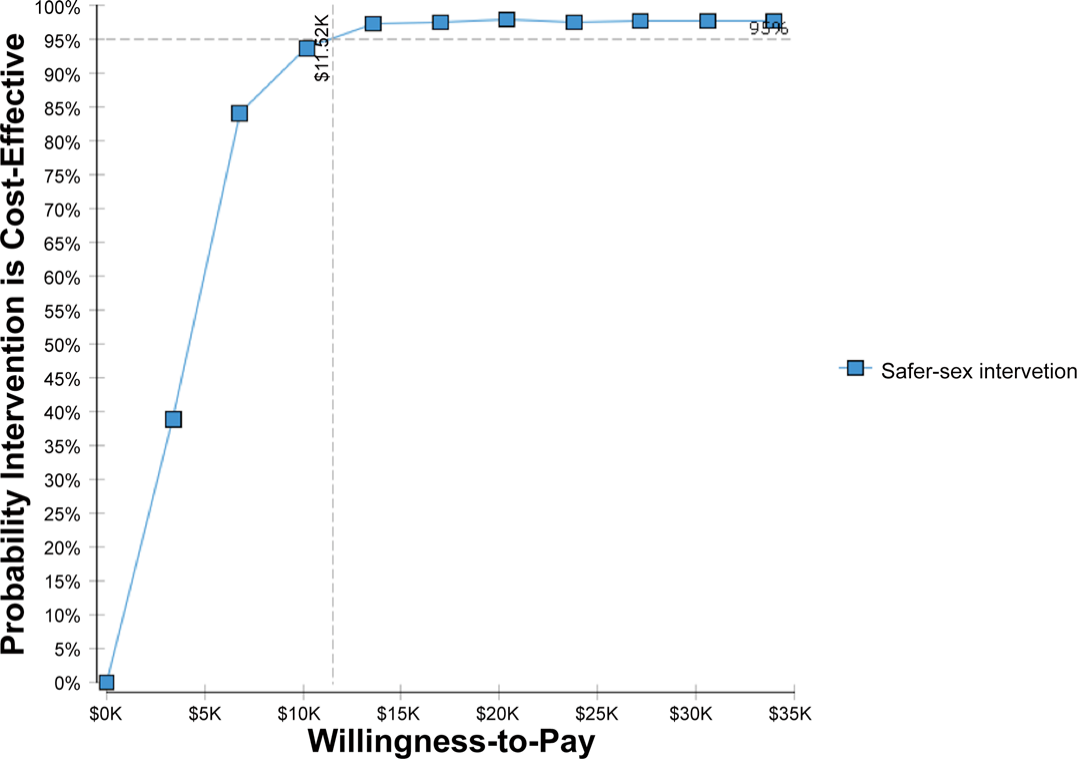
Cost-effectiveness acceptability curve for the combined interactive safer sex and didactic safer injection components of the Mujer Mas Segura intervention in Tijuana.

As shown in Fig 4 Results from the multivariate analysis show that for Tijuana, there is a 95% likelihood that the cost per QALY gained from the combined interactive safer sex and the didactic safe injection components of the Mujer Mas Segura intervention would cost less than US$11,522.22 equivalent to less than 3 times the GDP per capita in Mexico compared with the control group (didactic safer sex/safer injection MMS).[34] Multivariate analysis for the strategies combining the safer sex didactic components of the intervention (i.e. combined didactic/interactive components with the safer sex didactic component of the Mujer Mas Segura intervention) showed less than 20% likelihood of costing less than three times de GDP per capita in Mexico (data not shown).

## Discussion

Our analysis shows that the MMS intervention can be highly cost-effective in reducing HIV/STI risky behaviors among Mexican FSW in the context of on-going injection drug use, albeit mixed results. Our results suggest that in the context of limited reach or absent NEP, the interactive format of the safer injection combined with the interactive safer sex component of the MMS intervention can become cost-effective, but as syringe distribution at the community level is expanded, as seen in Tijuana during the MMS study period, the interactive safer injection format of the MMS intervention appears to become redundant with no added benefit.[16] In the main outcome MMS trial publication (Strathdee PloS ONE 2013) an expansion of the NEP was documented in TJ during the MMS trial, which was documented and assessed. The analysis of that paper showed that if CJ had scaled up NEPs to the extent documented in Tijuana, the effect if the safer injection intervention interactive component would have been dampened.[16]

Our findings from the cost-effectiveness of the interactive safer sex component of the intervention extend earlier findings from a previous economic evaluation of the Mujer Segura behavioral intervention.[3, 10] The interactive safer sex combined with the didactic safer injection components of the MMS behavioral intervention were found to be highly cost effective in both Tijuana and Cd. Juarez compared to administering the didactic version of both components. Additionally in Cd. Juarez, the combined interactive formats of the safer sex and safer injection interventions were shown to be cost-effective by further reducing unsafe injection practices among FSWs compared to those assigned to the interactive safer sex and didactic safer injection formats. In contrast, in Tijuana, the strategy combining both interactive formats of the MMS intervention was not found to be cost-effective, suggesting no improvement in self-efficacy in avoiding syringe sharing compared to FSW-IDUs assigned to the didactic safer-injection component of the MMS intervention. Our results were robust on multivariate sensitivity analysis for the combined interactive safer sex/didactic safer injection MMS intervention in both cities under all plausible scenarios.

In summary, we found that the MMS brief, interactive safer sex intervention combined with the didactic safer injection counseling session was highly cost-effective in reducing HIV/STI risks among FSWs in the context of ongoing injection drug use in Tijuana and Cd. Juarez. Our findings support previous studies that shows community level programs such as the scale up of NEP, pharmacy access to syringes or over the counter sale of syringes to be potentially more cost-effective than individual level interventions such as the more intensive interactive safer injection component of the MMS behavioral intervention, as shown in other settings.[35] This finding are plausible if HIV risks among FSWs who inject drugs are mostly driven by unsafe sex practices with male partners versus unsafe injection practices as some studies suggest.[36–38]. Under this scenario, this would explain the consistency of our study findings where the more intensive interactive safer sex intervention combined with the didactic safer injection components of the Mujer Mas Segura intervention remained cost-effective across two cities with reported differences in access to free condoms and sterile syringes among FSW-IDUs.[16]

Some limitations for our study include the fact that FSW-IDUs from Tijuana and Ciudad Juarez participating in the Mujer Mas Segura trial represented a sample at very high risk for HIV and STIs; thus, our study results might not be generalizable to other regions in Mexico. Although multivariate sensitivity analyses produced reliable confidence intervals, parameters used to model HIV progression were obtained from published reports from other populations (e.g. in the US and Africa), as additional data from Mexico were lacking.

Our findings have clear policy implications on the societal value of scaling up evidence based HIV prevention behavioral interventions at an individual level, such as the MMS, in combination with community level interventions such as NEP and condom distribution programs among FSW-IDUs in settings such as Tijuana and Cd. Juarez.[39, 40]

Recently, Mujer Segura was successfully implemented by a community-based organization in 12 cities across Mexico,[41] which suggests that this intervention could be brought to scale. Data from the MMS trial found a robust association between increased investment in NEPs and reduction in injection risk behaviors at an optimal cost-effectiveness level compared to providing the more intensive interactive safer injection component of the MMS behavioral intervention combined with the interactive safer sex component. These findings underscore the significant benefit of NEPs to reduce HIV risks among FSW-IDUs, and suggest that the interactive safer injection intervention is best reserved for circumstances where syringe access is poor (e.g., prisons) or is impeded by syringe prescription/possession laws. Unfortunately, since the MMS trial ended, Mexico’s support from the Global Fund ended, which has dramatically reduced sterile syringe availability in both cities and others across Mexico. A recent study in Tijuana suggests that two thirds of HIV infections among IDU, MSM, FSWs and their clients were phylogenetically unlinked, [38,40,41] which implies that there are now multiple, new introductions of HIV.[42] These findings should serve as a warning to policymakers that continued investments are needed to sustain evidence- based HIV prevention interventions such as MMS in the context of expanded NEPs before HIV incidence escalates in settings with pervasive injection drug use such as Tijuana and Cd. Juarez.

Additional supporting information for this analysis is available in the following website: http://figshare.com/preview/_preview/1606233#reserve_citation.
